# Towards a common core dataset for critical care: a registry-centered vision for global improvement

**DOI:** 10.62675/2965-2774.20260338

**Published:** 2026-03-26

**Authors:** Vrindha Pari, David Pilcher, Luigi Pisani

**Affiliations:** 1 University of Birmingham Institute of Applied Health Research Birmingham United Kingdom Institute of Applied Health Research, University of Birmingham - Birmingham, United Kingdom.; 2 The Australian and New Zealand Intensive Care Society Centre for Outcome and Resource Evaluation Prahan Victoria Australia Centre for Outcome and Resource Evaluation, The Australian and New Zealand Intensive Care Society - Prahan, Victoria, Australia.; 3 University of Bari "Aldo Moro" Section of Anesthesiology and Intensive Care Medicine Department of Precision-Regenerative Medicine and Jonic Area Bari Italy Department of Precision-Regenerative Medicine and Jonic Area, Section of Anesthesiology and Intensive Care Medicine, University of Bari "Aldo Moro" - Bari, Italy.

## THE EVOLVING LANDSCAPE OF CRITICAL CARE

Critical care, as a data-intensive field, involves a high volume of complex data generated by intensive care units (ICUs), transforming patient care into a data-driven science.^(1)^ The data collected is utilized in many ways, with quality improvement, benchmarking, and research playing an important part in interpreting and reporting this data. However, ICU data vary widely in the variables chosen, the definitions used, and the reporting formats. Varied geographic locations, health system contexts, infrastructure, historical evolution, and resource availability cause this variation. For example, the calculation of standardized mortality rates (SMRs) differs depending on which scores are used and their definitions. The European and South American registries use Acute Physiology and Chronic Health Evaluation (APACHE) or Simplified Acute Physiology Score (SAPS). In contrast, other low- and middle-income countries (LMICs) use locally developed models such as EtropICs.^(2)^ This lack of standardization in outcome measures reduces the potential of the data to be used for benchmarking, comparisons, and generalizable research. These are just a few of the many key reasons for systematically collecting data in electronic health records (EHRs) and/or clinical quality registries.^(3,4)^

The importance of large-scale benchmarking and quality improvement initiatives can be addressed in many ways. One way to address this and navigate the issue of variable datasets is to create common core datasets (CCDS). A CCDS is a list of variables, measures, and their definitions that are standardized and implemented in the same way across the data registries or EHRs that adopt this common dataset.^(5)^ Data registries rather than EHRs are particularly well suited for this, as their architectures are usually designed with inherent adaptability. While EHRs systems are often limited by local configurations and rigid structures, data registries are generally built to adapt and evolve in response to the users’ needs. Registries support benchmarking within and between ICUs as part of their design.^(4)^

## THE PROMISE OF INTENSIVE CARE UNIT REGISTRIES - FROM DATA COLLECTION TO ACTION

In an effort to enable large-scale, systematic data collection, many regional and national data registries have been established worldwide. Intensive care unit registries are a powerful platform for implementation research, enabling real-time recruitment, intervention evaluation, and the development of hybrid designs that build local capacity.^(6,7)^ More than 3,000 ICUs worldwide participate in ICU registries across more than 30 countries, covering around 40,000 ICU beds.^(2,8)^ These ICU beds are representative of ICU registries from across 33 countries, with a majority LMIC representation from Asia, Africa, and South America, but with roughly half of the registries from ICUs in South America.^(2)^ Notable examples include European registries like Intensive Care National Audit & Research Centre (ICNARC), National Intensive Care Evaluation (NICE), and CUB-réa; The Asia-Pacific region has registries like the Australian and New Zealand Intensive Care Society (ANZICS,) Centre for Outcome and Resource Evaluation (CORE) and the Japanese Intensive care PAtient Database (JIPAD); Latin American initiatives such as SATI-Q and Ibero-American Intensive Care Units Registry; and the Critical Care Asia-Africa network (CCAA) supporting national registries in more than ten African and Asian countries.^(9,10)^ The ICNARC registry data have informed country-wide staffing standards and performance benchmarking across the National Health Service (NHS) in the UK. Similarly, CCAA has provided ICUs in resource-constrained settings a platform to identify improvement priorities, track outcomes, and standardize processes via multinational benchmarking dashboards.

## THE CASE FOR CREATION OF COMMON CORE DATASETS – A DUAL-EDGED SWORD

There are existing initiatives that have advanced standardization, but a universally adopted common core dataset remains elusive. Localized efforts toward common data sets/elements, such as AO Spine RECODE-DCM for degenerative cervical myelopathy and SCCM's critical care data dictionaries (C2D2),^(5,9)^ demonstrate successful standardization within specific domains. Existing diagnostic terminology lists, such as Systematized Nomenclature of Medicine Clinical Terms (SNOMED CT) and the International Classification of Diseases (ICD) 10/1, share common terminology, providing scope for standardization. Similarly, severity-of-illness scores such as APACHE IV and SAPS are widely used in such lists. But these still lack standardization when viewed through global benchmarking and outcome comparisons. Key barriers to global adoption include substantial variations in local clinical priorities and practices, funding constraints that make implementation challenging (particularly in LMICs), and complex requirements for integration into existing systems. As a result, many settings adopt only selected elements or struggle to maintain ongoing harmonization, thereby limiting the broad global comparability these initiatives aim to achieve.^(11)^ This heterogeneity and lack of standardization give rise to many challenges (e.g., data quality, completeness etc.) that are further influenced by the differences in resource availability and infrastructure.^(8,9)^ There is ultimately a lack of a single initiative or an entity that has the capacity to facilitate and/or implement a truly common core dataset.

There is increasing impetus to share data to drive improvement in critical care, stemming from the coronavirus disease (COVID-19) pandemic. Many publicly available critical care public health databases (e.g., American-based databases such as Medical Information Mart for Intensive Care (MIMIC) and the eICU Collaborative Research Database (eICU-CRD), and a freely accessible European database such as the Amsterdam University Medical Center Database (AmsterdamUMCdb)^(5)^ have emerged. This increasing interest in data sharing and willingness in international collaboration presents unique opportunities for global initiatives like Linking Of Global Intensive Care (LOGIC)^(10,12)^ to further this pursuit of standardization and data sharing. Standardization of data through the development of a common core dataset can provide real-time feedback mechanisms that improve motivation and support, in turn improving care, potentially bridging the gap between data from high- and low-income countries,, and providing equitable insights.^(13)^ For example, the ANZICS CORE registry introduced near-real-time unit-level reporting, allowing ICUs to identify performance outliers and rapidly implement changes that reduced mortality and improved sepsis management outcomes. Real-time feedback speeds up the identification of care gaps and supports efficient, data-driven improvements, enabling continuous monitoring and empowering providers to act quickly. Linking ICU registries with nationwide administrative databases is increasingly a reality, enabling the identification of inequalities and potential areas for improvement.^(14)^ This level of promptness fosters equitable care by giving both high- and low-income settings access to timely quality insights, reducing disparities in critical care outcomes worldwide. Essentially, real-time feedback transforms data into actionable knowledge that narrows global gaps in critical care quality.

## A LOGICAL SOLUTION

Linking Of Global Intensive Care (LOGIC) is an international network of critical care registries that currently includes 13 registries from over 18 countries.^(12)^ Its potential to facilitate the creation of CCDS is apparent by its decentralized, data sovereignty-protecting model, which reduces privacy barriers while creating a truly open and inclusive international benchmarking initiative.^(12)^ LOGIC manages data sovereignty and data privacy issues by ensuring that individual patient data stays within the contributing registries, only aggregated, national-level datasets are shared for benchmarking, thus avoiding the regulatory and privacy challenges typically associated with cross-border data transfer. By convening diverse stakeholders from various countries and contexts, LOGIC can define a pragmatic CCDS through systematic consensus-building, prioritizing feasibility and relevance for LMICs. This platform can also serve as a data-mapping center, standardizing data before aggregation and reducing the burden on individual sites. Ultimately, LOGIC can serve as a knowledge hub providing best practices for CCDS implementation, which is important for LMICs.

However, even with agreed definitions, interpretations in the real world can vary. Variations in resources across LMICs could hinder data integration and reporting consistency, requiring continuous quality management. Sharing patient information across borders would also require navigating diverse international data protection laws (such as General Data Protection Regulation [GDPR], Health Insurance Portability and Accountability Act [HIPAA], and other national regulations).^(15)^ Maintaining such an international initiative would require robust governance for its growth and development.

## TOWARD A UNIFIED FRAMEWORK – A ROADMAP FOR STANDARDIZATION

**Figure 1:**
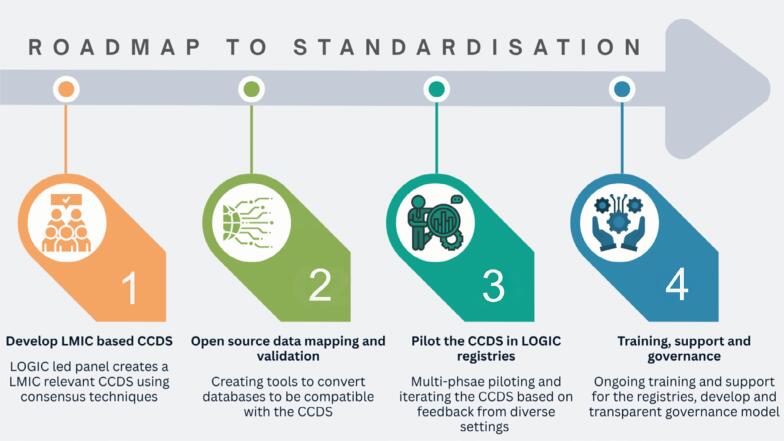
Visual map of roadmap towards creating Common Core Datasets.

Achieving this vision requires a phased roadmap:

– Phase 0 - 2024-25 (completed): A LOGIC lead global mapping of existing core datasets across global ICU registries - Global Registry ICU datasets (GRID) have been completed and are being published.
– Phase 1- 2026: a LOGIC led expert panel to finalize a clinically relevant and feasible registry-centered CCDS for critical care that would be actionable in LMICs using consensus techniques. This phase would leverage existing core datasets, such as C2D2.
– Phase 2 - Jan to Jun 2027: Developing open-source data mapping and validation tools to facilitate converting local datasets compatible with the CCDS.
– Phase 3 - Jun to Dec 2027: A planned multi-phase piloting of the CCDS in LOGIC registries representing diverse settings and iterating the CCDS based on feedback.
– Phase 4 - 2028: Establish an ongoing training and support system for data collectors and clinicians, and establish a transparent governance model - one involving diverse LMIC representation in positions of power and decision making, and consistent stakeholder involvement for feedback on the governing processes would support the evolution of the CCDS and advocate for supportive policies (Figure 1).

LMIC - low- and middle-income countries; CCDS - create common core datasets; LOGIC - Linking Of Global Intensive Care.

Ultimately, the creation of a common core dataset for critical care is a foundational requirement to meet the global need for high-quality, data-driven quality improvement and effective benchmarking. A registry-centered approach, building upon existing initiatives like LOGIC, can revolutionize global critical care by providing the space for robust international benchmarking, international research, and quality improvement. This ambitious vision is achievable with continuous collaboration and planned investment from the stakeholders, with the ultimate aim of identifying and tackling inequalities. This will pave the way for an integrated and influential critical care ecosystem worldwide.

## Data Availability

The contents underlying the research text are included in the manuscript.
